# Increased risk of thyroid diseases in patients with systemic lupus erythematosus: A nationwide population-based Study in Korea

**DOI:** 10.1371/journal.pone.0179088

**Published:** 2017-06-27

**Authors:** Jae-Seung Yun, Jung Min Bae, Ki-Jo Kim, Yu Seok Jung, Gyong Moon Kim, Hyung-Rae Kim, Jun-Seok Lee, Seung-Hyun Ko, Seon-Ah Cha, Yu-Bae Ahn

**Affiliations:** 1 Division of Endocrinology and Metabolism, Department of Internal Medicine, St. Vincent’s Hospital, College of Medicine, The Catholic University of Korea, Suwon, Korea; 2 Department of Dermatology, St. Vincent’s Hospital, College of Medicine, The Catholic University of Korea, Suwon, Korea; 3 Division of Rheumatology, Department of Internal Medicine, St. Vincent’s Hospital, College of Medicine, The Catholic University of Korea, Suwon, Korea; National Cancer Institute, UNITED STATES

## Abstract

We investigated the association between autoimmune thyroid disease and systemic lupus erythematosus (SLE) using nationwide insurance claims data for the entire Korean population. Claims data for the period 2009–2013 were retrieved from the National Health Insurance System database. SLE and thyroid disease were identified using the International Classification of Diseases codes and medication information. Logistic regression analyses were used to evaluate the association between SLE and thyroid disease. The study used records from 17,495 patients with SLE and 52,485 age- and sex-matched control subjects. A greater prevalence of Graves’ disease (0.94% vs. 0.46%, *P* < 0.001), Hashimoto’s thyroiditis (2.68% vs. 0.80%, *P* < 0.001), and thyroid cancer (1.81% vs. 1.30%, *P* < 0.001) was observed in SLE patients than in control subjects. Multivariate regression analyses demonstrated that SLE was significantly associated with an increased risk of both autoimmune thyroid disease and thyroid cancer **(**Graves’ disease: odds ratio [OR] 2.07, 95% confidence interval [CI] 1.70–2.53; Hashimoto’s thyroiditis: OR 3.42, 95% CI 3.00–3.91; thyroid cancer: OR 1.40, 95% CI 1.22–1.60). Age- and sex- stratified analyses revealed that the risk of autoimmune thyroid disease in SLE patients was increased for all age groups and the female group. An association between thyroid cancer and SLE was identified only in the 20- to 59-year-old age group and in the female group. Using a large population-based study, we demonstrated that patients with SLE are at a greater risk of developing thyroid disease than matched control individuals.

## Introduction

Systemic lupus erythematosus (SLE) is an autoimmune disease characterized by disturbances in the immune response and autoantibody production that lead to multi-system organ damage and dysfunction [1]. SLE is well known for its immunologic diversity and often presents with a broad spectrum of clinical and immunologic manifestations that include neurologic, hematologic, and cardiovascular symptoms, or even cancer [2, 3]. Further, the risk of death for SLE patients is three-fold greater than that for the general population [3, 4]. The high morbidity and mortality associated with SLE only adds to the disease burden and markedly reduces the quality of life [5].

Autoimmune thyroid disease (AITD) is a well-known, organ-specific autoimmune disorder that is associated with many non-specific autoimmune diseases such as rheumatoid arthritis, Sjögren’s syndrome, and SLE [6]. AITD occurs as a result of a T-cell-mediated autoimmune response characterized by diffuse lymphocytic infiltration of the thyroid gland. The possibility that a significant association exists between SLE and thyroid disease has been consistently suggested since the first case of SLE and Hashimoto’s thyroiditis was described 50 years ago [7]; however, whether SLE is associated with a greater incidence of thyroid disease is still unclear.

Several studies have shown a higher prevalence of thyroid disease and anti-thyroid antibodies in SLE patients than in the general population, even in those without overt clinical thyroid disease [8–11]. Most studies have consistently demonstrated that the prevalence of hypothyroidism in SLE patients is higher than that of the general population [9, 12]. In contrast, studies on the prevalence of Graves’ disease in SLE patients show considerable variation. Although two case-control studies showed a higher prevalence of hyperthyroidism, including Graves’ disease and subclinical hyperthyroidism, in SLE patients than in healthy control subjects [9, 13], other studies report no such increase in the prevalence of hyperthyroidism in SLE patients [8, 12, 14]. An important limitation in some of these earlier studies is the small sample size, probably because of the low numbers of SLE patients. Further, some studies either had no cases of hyperthyroidism in SLE patients or included only a few such cases [10, 12]. Even though more recent studies have investigated the relationship between thyroid disease and SLE using data from large populations, they also provide inconsistent and conflicting results. A Taiwanese retrospective nationwide cohort study showed that SLE patients had a significantly lower rate of thyroid disease [15], whereas another recent study in Israel reported that both hyper- and hypothyroidism were associated with SLE [13, 16].

The purpose of this study was to investigate a possible association between SLE and thyroid disease using insurance claims data for the entire population of Korea.

## Materials and methods

### Study design and data sources

We conducted a nationwide cross-sectional study using the Korean National Health Insurance (NHI) Claims Database of the Health Insurance Review Agency for the period between 2009 and 2013. This database includes all claims data provided by the NHI program and the Medical Aid program [17]. The Korean NHI program is a compulsory social insurance scheme that covers approximately 97% of the population; the remaining 3% are protected under the Medical Aid program [18]. The database used in this study consists of anonymized data for research purposes. The Catholic Medical Center Ethics Committee approved this study (Institutional Review Board approval number VC16EISE0065).

### Study population

Patients were categorized as part of the SLE group if they had been treated under M32 of the International Classification of Diseases 10th revision (ICD-10) code at least five times between 2009 and 2013. Subjects for the control group were randomly selected from an initial screening of all patients who had undergone an appendectomy or hemorrhoid surgery without a diagnosis of SLE during the same period, such that there were three age- and sex-matched controls per SLE patient.

### Definitions of thyroid diseases

Both ICD-10 codes and thyroid medication prescriptions were used to define AITD. Patients with Graves’ disease were defined as those diagnosed with hyperthyroidism (E05.0, E05.00, E05.01, E05.8, E05.9) and prescribed anti-thyroid drugs, including methimazole, propylthiouracil, and carbimazole, for at least 60 days. Those with toxic thyroid nodules (E05.1), toxic multinodular goiter (E05.2), acute thyroiditis (E06.0), and subacute thyroiditis (E06.1) were not included in the study. Patients with Hashimoto’s thyroiditis were defined as those diagnosed with autoimmune thyroiditis (E06.3, E06.9) and prescribed thyroid hormone replacement for at least 60 days. Patients with thyroid cancer were defined as those treated at least five times under the ICD-10 code for thyroid cancer (C73).

### Subgroup analyses

Subgroup analyses were performed according to age (≤19, 20–39, 40–59, ≥60 years) and sex to assess their effects on the risk of thyroid disease in SLE patients compared with control subjects.

### Statistical analyses

Prevalence was calculated as the number of cases divided by the total population in 2013. Descriptive statistics are presented as frequencies and percentages. Categorical variables are expressed as percentages and were compared using the χ^2^ test. Both crude and age- and sex-adjusted odds ratios (ORs) with 95% confidence intervals (CIs) were calculated, and a logistic regression analysis was used to determine the association between SLE and thyroid disease. Sensitivity analyses after additional adjustment for insurance type (NHI or Medical Aid) were performed to ensure the robustness of the results. All data analyses were carried out using SAS software (version 9.4; SAS Institute, Cary, NC, USA).

## Results

### Characteristics of the study population

We identified and evaluated the records of 17,495 patients with SLE and 52,485 age- and sex-matched control subjects without SLE for data analyses (Table 1). The prevalence of SLE was 35.0 cases per 100,000 persons. In both SLE and control groups, the mean age was 40.0 ± 13.9 years (range, 4–95 years). The majority were women (90.5%), and the population density was highest in the 30- to 39-year-old age group.

**Table 1:** Descriptive characteristics of the study population.

	SLE patients, n (%)	Controls, n (%)	*P*-value
**Total**	17,495	52,485	
**Age group, years**			0.980
0–19	1,149 (6.5)	3,425 (6.5)	
20–39	7,780 (44.5)	23,265 (44.3)	
40–59	7,029 (40.2)	21,203 (40.4)	
≥60	1,537 (8.8)	4,592 (8.8)	
**Sex**			0.999
Male	1,669 (9.5)	5,007 (9.5)	
Female	15,826 (90.5)	47,478 (90.5)	
**Insurance type**			<0.001
Health insurance	16,284 (93.1)	51,930 (98.9)	
Medical aid	1,211(6.9)	555 (1.1)	

SLE, systemic lupus erythematosus.

### AITDs in patients with SLE

The prevalence of Graves’ disease and Hashimoto’s thyroiditis was 0.94% (165/17,495) and 2.68% (468/17,495), respectively. After adjusting for age and sex, patients with SLE exhibited a greater risk for Graves’ disease (OR 2.07, 95% CI 1.70–2.53, *P* < 0.001) and Hashimoto’s thyroiditis (OR 3.42, 95% CI 3.00–3.91, *P* < 0.001) than control subjects.

### Thyroid cancer in patients with SLE

The prevalence of thyroid cancer in SLE patients was 1.81% (316/17,495), which was significantly higher than that of the control group [1.30% (684/52485); *P* < 0.001]. After adjustment for age and sex, SLE patients had a significantly higher risk of thyroid cancer (OR 1.40, 95% CI 1.22–1.60, *P* < 0.001).

### Subgroup analyses according to age and sex

**Fig 1:**
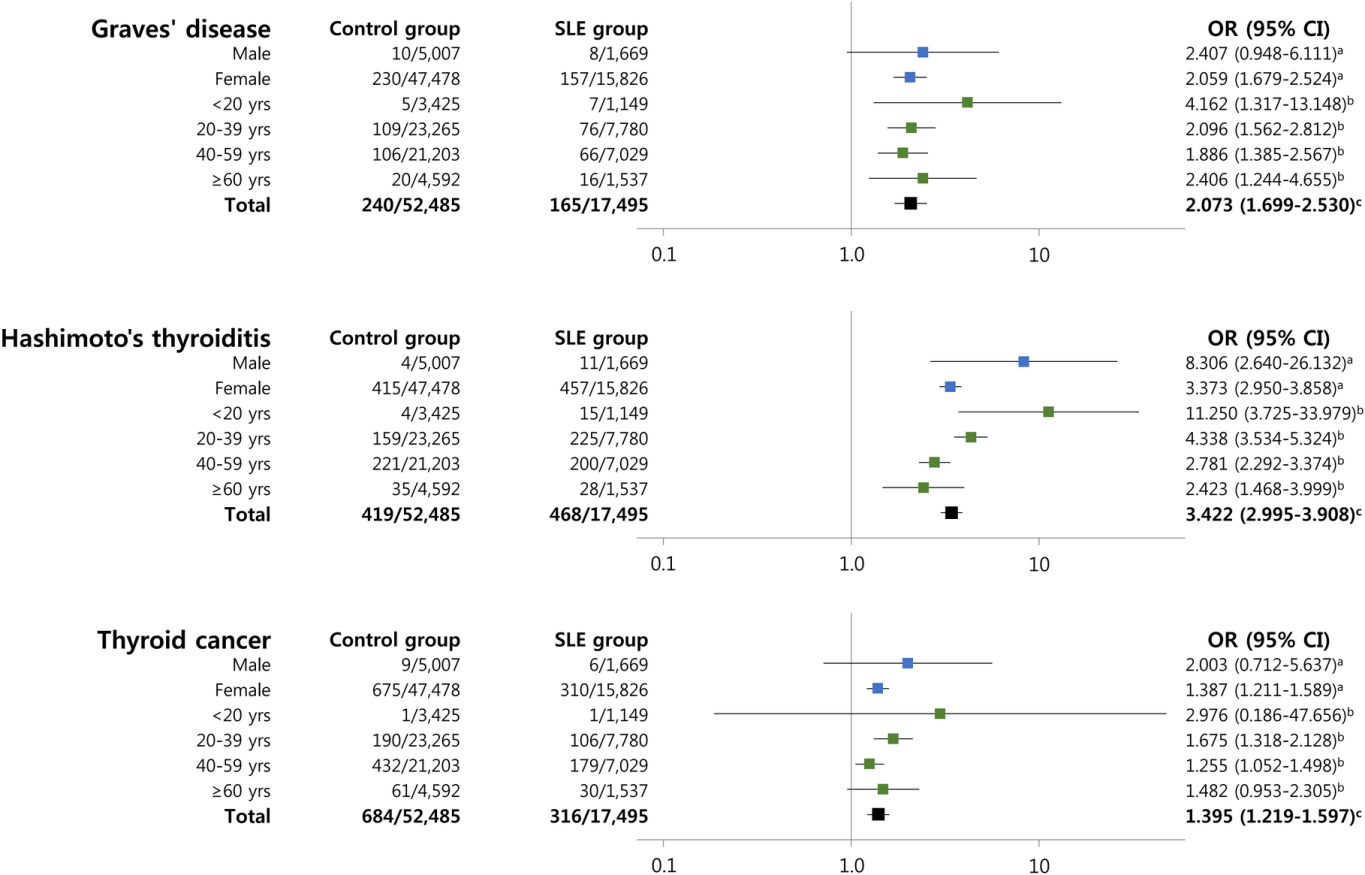
Analyses stratified according to age and sex for evaluating the association between SLE and thyroid disease. ^a^Adjusted for age. ^b^Adjusted for sex. ^c^Adjusted for age and sex.

The OR for thyroid disease was higher in female SLE patients than in control subjects, whereas the risk of Hashimoto’s thyroiditis was significantly elevated in male SLE patients. In contrast, there were no significant differences between control subjects and SLE patients with respect to the prevalence of Graves’ disease and thyroid cancer in men (Fig 1).

Fig 1 shows the relationship between SLE and thyroid disease when stratified by age and illustrates that the prevalence of Hashimoto’s thyroiditis increased with age in SLE patients. The association between thyroid cancer and SLE was weaker in the ≥60-year-old age group than in the 21- to 39-year-old age group, whereas the association between AITD and SLE was much stronger in the <40-year-old age groups. In the <20-year-old age group, although the prevalence of thyroid disease was very low, Hashimoto’s thyroiditis (OR 11.25, 95% CI 3.73–33.98, *P* < 0.001) and Graves’ disease (OR 4.16, 95% CI 1.32–13.15, *P* = 0.015) showed a significant association with SLE. Sensitivity analysis after additional adjustment for insurance type did not alter the significance of the ORs (Table 2).

**Table 2:** Association between SLE and thyroid disease.

	n (%)	Univariable analysis		Multivariable analysis		Sensitivity analysis	
		Crude OR (95% CI)	*P*-value	Adjusted OR (95% CI)^a^	*P*-value	Adjusted OR (95% CI)^b^	*P*-value
**Grave’s disease**							
Control	240 (0.46)	Reference		Reference		Reference	
SLE patients	165 (0.94)	2.07 (1.69–2.54)	<0.001	2.07 (1.70–2.53)	<0.001	2.01 (1.64–2.46)	<0.001
**Hashimoto’s thyroiditis**							
Control	419 (0.80)	Reference		Reference		Reference	
SLE patients	468 (2.68)	3.42 (2.99–3.90)	<0.001	3.42 (3.00–3.91)	<0.001	3.50 (3.06–4.00)	<0.001
**Thyroid cancer**							
Control	684 (1.30)	Reference		Reference		Reference	
SLE patients	316 (1.81)	1.39 (1.22–1.59)	<0.001	1.40 (1.22–1.60)	<0.001	1.42(1.24–1.63)	<0.001

SLE, systemic lupus erythematosus; OR, odds ratio; CI, confidence interval.

^a^Adjusted for age and sex.

^b^Adjusted for age, sex, and insurance type.

## Discussion

The present study demonstrates that SLE patients have a higher risk of developing AITD and thyroid cancer; this association was especially pronounced in women and in the <40-year-old age groups.

As we assessed the relationship between AITD and SLE using the national Korean population database, the total numbers of patients with SLE and with each thyroid disease were much greater than values reported in previous studies, and we show that the risk of both Graves’ disease and Hashimoto’s thyroiditis was significantly higher in SLE patients than in the control subjects. We also show a greater prevalence of Hashimoto’s thyroiditis than Graves’ disease in SLE patients. Moreover, although both Graves’ disease and Hashimoto’s thyroiditis were more highly associated with SLE in women, only Hashimoto’s thyroiditis was associated with SLE in men despite the lower prevalence of AITD in men. The differences in prevalence and association data between those reported here and previous reports may be attributable to the fact that the definition of thyroid disease in our study differs from that of the earlier studies. We defined thyroid disease as occurring in those patients who had been prescribed thyroid disease-related medication in order to be able to identify cases that would help focus on the relationship between clinically overt thyroid disease and SLE. This definition was based on the results from a previous Korean nationwide survey that showed that anti-thyroid drugs were the preferred treatment regimen for first-time and recurrent Graves’ disease and that most patients chose either anti-thyroid drugs (97.1%) or radioactive iodine (2.9%) rather than surgery (0.0%) [19], notwithstanding the fact that thyroid hormone replacement is essential for treating overt clinical hypothyroidism [20].

Several possible hypotheses can explain the association between SLE and thyroid disease. Both SLE and AITD are caused by a loss of immunological tolerance and show exaggerated cellular and humoral immune responses [21, 22]. The activation of both the autoreactive T cells and the polyclonal B cells in SLE can target components of the thyroid gland and induce AITD. A shared genetic predisposition has also been suggested as a possible explanation because one study on families with SLE and concomitant AITD showed the presence of an AITD susceptibility gene in the 5q14.3-q15 locus, which is also the major susceptibility locus for SLE [23]. Another study has suggested a role for HLA-B8 and DR3 in patients with SLE and Hashimoto’s thyroiditis [24]. Recent genome-wide association studies revealed that certain gene loci, including the R620W polymorphism of the protein tyrosine phosphatase PTPN22 and the interferon induced with helicase C domain 1 (IFIH1) rs1990760 polymorphism, are associated with susceptibility to multiple autoimmune diseases including Graves’ disease, Hashimoto’s thyroiditis, and SLE [25, 26]. Infectious agents and smoking are other possible environmental triggers of autoimmune diseases [27].

The risk of malignancy in SLE patients is another important issue. The association between autoimmune disease and cancer has been under study for over a decade [28]. Possible commonly suggested pathways linking SLE and increased risk of cancer include impairment of the immune system to identify and destroy early-stage tumor by the use of immunosuppressive drugs, that is, impaired immune surveillance [28, 29]. Previous reports show that cancer risk has an inverse association with age in SLE patients, which is different from the general population [30]. This inverse association might be because of the higher degree of clinical severity in the early stage of SLE, and the use of more aggressive immunosuppressive agents may contribute to the impaired immune surveillance and the progression to clinical cancer at this stage. Subgroup analysis of studies and a meta-analysis of several studies report a higher frequency and risk of thyroid cancer in SLE patients [30–33]. The mechanism by which the thyroid cancer risk is increased in SLE patients is still unclear. Proposed explanations for the association between thyroid cancer and SLE include chronic inflammation due to autoimmunity, shared genetics, environmental links, and medications [34]. Of these, thyroid autoimmunity and chronic thyroid inflammation are reasonable mechanisms that link thyroid cancer with SLE [35, 36]. This hypothesis is underpinned by the fact that a greater proportion of SLE patients with thyroid cancer had thyroid autoimmunity compared with those without thyroid cancer [37].

Sex differences are specific characteristics of SLE with respect to clinical manifestations and outcomes [38, 39]. Previous studies showed that the manifestations of alopecia and arthritis were found less frequently in male SLE patients than in female SLE patients, and the incidence of renal disease and hematologic involvement were also more frequent. In addition, higher mortality rates have been reported in male SLE patients [40, 41]. However, there are very few studies about sex differences in the incidence of thyroid disease among SLE patients. Watad et al. reported that the risk of hyper- and hypothyroidism are increased in male as well as in female SLE patients [13, 16]. Regarding cancer, both female and male SLE patients had an increased standardized incidence ratio of overall cancer compared with the general population [30, 42]. However, to our best knowledge, there is no known report of a difference between male and female SLE patients for the risk of thyroid cancer. In our study, Hashimoto’s thyroiditis was found to be significantly increased in both male and female SLE patients, although Graves’ disease and thyroid cancer did not show a statistically significant increase in their ORs. The very low prevalence of Graves’ disease and thyroid cancer in male SLE patients makes it difficult to analyze the risk of thyroid disease in these patients. Further studies are needed to determine whether the difference in the risk of thyroid disease is due to the sex-specific characteristics or to sex differences in disease activity or drug use in SLE patients.

Subgroup analyses in our study revealed that the risk of Hashimoto’s thyroiditis in SLE patients was significantly higher in the <40-year-old age groups than in the ≥40-year-old age groups, whereas the prevalence of AITD and thyroid cancer in the control group was highest in the 40- to 59-year-old age group [30]. This finding is comparable with an earlier nationwide study in Israel that analyzed the association between SLE and hypothyroidism [16]. One possible explanation for the increased risk of thyroid disease in young SLE patients might be the relatively higher disease activity in these patients [43, 44]. Nevertheless, this association between SLE activity and thyroid disease shown by previous studies is still controversial owing to limitations in study design and participant numbers, making it difficult to verify their conclusions [45, 46]. Also, as mentioned above, the use of aggressive immunosuppressive agents during the early stages of the disease can contribute to the later development of overt thyroiditis [30, 47], and the maximum inflammatory response in SLE patients would be expected after the reduction in immunosuppressive therapy. Thus, SLE patients can develop thyroiditis after the cessation of immunosuppressive therapy. In addition, the cytotoxic effects of the long-term accumulation and aggressive use of immunosuppressive agents may also contribute to the onset of thyroid disease. Lastly, there is the possibility that a shared genetic susceptibility between SLE and AITD might play a role in triggering the early development of autoimmune disease in SLE patients, although no clear evidence of this has been demonstrated yet [48, 49].

There are several limitations to the present study. First, as this study was based on a medical claims database, there is a possibility of incorrect diagnoses. However, SLE is classified as a rare and incurable disease in Korea, and the government supports medical costs for all SLE patients assigned an SLE code (M32). As the assignment of SLE diagnostic codes is strictly managed by review and evaluation by the Health Insurance Review Agency, the accuracy of SLE claims data is higher than that of other diseases, and this has been validated by an earlier study [50]. The second limitation is that thyroid disease was diagnosed only by claims data. No detailed information on thyroid autoantibodies, histologic subtypes of thyroid cancer, thyroid ultrasonography findings, or the disease severity and lifestyle of each subject was available; therefore, the possibility of selection or confounding bias cannot be ruled out. To avoid these biases, we categorized patients as having thyroid disease only if they had been prescribed medicine for Graves’ disease or Hashimoto’s thyroiditis or had at least four physicians document that thyroid cancer was their primary diagnosis. Regarding the pathologic subtypes of thyroid cancer, a previous analysis from the Korea Central Cancer Registry showed that papillary thyroid cancer accounted for 97.2% of total thyroid cancer [51]. Therefore, although we did not subdivide thyroid cancer according to pathologic type, most of the thyroid cancer in this study was considered to be included in papillary thyroid cancer. Nevertheless, the strength of this study lies in the fact that all relevant medical data for the Korean population were included in the analyses. Furthermore, to the best of our knowledge, this is the largest population-based survey on the association between SLE and thyroid disease to date.

In conclusion, we demonstrate that, in the Korean population, SLE patients have a high risk of developing AITD and thyroid cancer. In particular, the risk of thyroid disease in SLE patients appeared to be higher among patients in the younger age group compared with other age groups. Further studies are necessary to delineate the exact mechanisms of and factors affecting this association.
